# Barriers and facilitators to implementing the CURE stop smoking project: a qualitative study

**DOI:** 10.1186/s12913-021-06504-2

**Published:** 2021-05-20

**Authors:** Angela Wearn, Anna Haste, Catherine Haighton, Verity Mallion, Angela M. Rodrigues

**Affiliations:** 1 Department of Psychology, Northumbria University, Northumberland Building, Newcastle upon Tyne, NE1 8ST UK; 2 Population Health Sciences Institute, Newcastle University, Newcastle upon Tyne, NE1 4AX UK; 3 Fuse: UKCRC Centre for Translational Research in Public Health, Newcastle upon Tyne, UK; 4 Department of Psychology, School of Social Sciences, Humanities and Law, Teesside University, Middlesbrough, TS1 3BX UK; 5 Department of Social Work, Education and Community Wellbeing, Northumbria University, Coach lane Campus West, Newcastle upon Tyne, NE7 7XA UK; 6 Public Health England, Wellington House, 133-155 Waterloo Road, London, SE1 8UG UK

**Keywords:** Behaviour change, Implementation, Smoking cessation, Tobacco dependence, Theoretical domains framework, COM-B model

## Abstract

**Background:**

The Conversation, Understand, Replace, Experts and evidence-based treatment (CURE) project aims to provide a comprehensive offer of both pharmacotherapy and specialist support for tobacco dependence to all smokers admitted to hospital and after discharge. CURE was recently piloted within a single trust in Greater Manchester, with preliminary evidence suggesting this intervention may be successful in improving patient outcomes. Plans are currently underway to pilot a model based upon CURE in other sites across England. To inform implementation, we conducted a qualitative study, which aimed to identify factors influencing healthcare professionals’ implementation behaviour within the pilot site.

**Methods:**

Individual, semi-structured telephone interviews were conducted with 10 purposively sampled health professionals involved in the delivery and implementation of the CURE project pilot. Topic guides were informed by the Theoretical Domains Framework (TDF). Transcripts were analysed in line with the framework method, with data coded to TDF domains to highlight important areas of influence and then mapped to the COM-B to support future intervention development.

**Results:**

Eight TDF domains were identified as important areas influencing CURE implementation; ‘environmental context and resources’ (physical opportunity), ‘social influence’ (social opportunity), ‘goals’, ‘professional role and identity’ and ‘beliefs about consequences’ (reflective motivation), ‘reinforcement’ (automatic motivation), ‘skills’ and ‘knowledge’ (psychological capability). Most domains had the potential to both hinder and/or facilitate implementation, with the exception of ‘beliefs about consequences’ and ‘knowledge’, which were highlighted as facilitators of CURE. Participants suggested that ‘environmental context and resources’ was the most important factor influencing implementation; with barriers most often related to challenges integrating into the wider healthcare context.

**Conclusions:**

This qualitative study identified multi-level barriers and facilitators to CURE implementation. The use of theoretical frameworks allowed for the identification of domains known to influence behaviour change, and thus can be taken forward to develop targeted interventions to support future service implementation. Future work should focus on discussing these findings with a broad range of stakeholders, to ensure resultant intervention strategies are feasible and practicable within a healthcare context. These findings complement wider evaluative work to support nationwide roll out of NHS funded tobacco dependence treatment services in acute care trusts.

**Supplementary Information:**

The online version contains supplementary material available at 10.1186/s12913-021-06504-2.

## Introduction

A number of key strategic documents including the Prevention Green Paper [1], the Tobacco Control Plan for England [2], the NHS Long Term Plan [3], and the Public Health England (PHE) Strategy [4] have outlined a commitment to reduce smoking rates and help England become a smoke-free society by 2030. The provision of NHS-funded tobacco treatment in secondary care has been highlighted as a key strategy for fulfilling these commitments [1–5]. However, the National Smoking Cessation audit [5] suggests greater improvement and provision of inpatient treatment is needed. For example, just under 1 in 4 hospital patients were not asked if they smoked,1 in 2 of those who smoked were not asked if they would like to quit and just 1 in 8 smokers were referred for hospital- or community-based treatment for their tobacco dependence.

To address this treatment gap, the Conversation, Understand, Replace, Experts and evidence-based treatments (CURE) project was developed within an NHS trust within Greater Manchester secondary care, based upon a successful approach to smoking cessation implemented within Ottawa, Canada [6]. The Ottawa model for smoking cessation (OMSC) focuses on the systematic identification and documentation of all smokers admitted to secondary care, and the provision of both pharmacotherapy and behavioural support to treat tobacco dependence. Patients are then attached to ongoing community follow-up post-discharge [6]. Aligned with this approach, the CURE project aims for cultural change within the secondary care context, encouraging healthcare providers to view tobacco dependence as a disease to be treated, rather than a lifestyle choice [7, 8]. In line with the OMSC, both existing secondary-care staff and a specialist nursing team deliver different aspects of the intervention. Within 24 h of admission, all inpatients are screened for smoking status by the admitting team. ‘Active’ smokers are offered brief advice to quit alongside Nicotine Replacement Therapy (NRT) and/or varenicline in line with their level of addiction. Smokers are also referred to the core team of CURE specialist stop smoking practitioners for ongoing pharmacotherapy and behavioural advice and support. This support is inclusive of follow-up appointments at 2, 4- and 12-weeks post-discharge.

The CURE project appears both feasible and effective [6, 7]. Although only in the early stages, preliminary evidence from the CURE project pilot highlights 22% of smokers admitted during the pilot phase reported abstinence at 12 weeks post discharge [7]. Evaluation of the OMSC suggests this model of smoking cessation may also be successful in improving health outcomes more broadly [6]. Alongside an increased likelihood of giving up smoking, outcomes included reduced number of visits to Accident and Emergency, reduced hospital readmissions and reduced mortality [6]. In terms of feasibility, 92% of adult admissions were successfully screened for smoking during the CURE project pilot phase, 96% of these smokers were provided with brief advice, 66% were prescribed pharmacotherapy to support their quit attempts and 61% completed inpatient behavioural interventions. Given the range of positive outcomes observed within Greater Manchester and Canada, national roll out of a model based on the OMSC and CURE is planned by 2023/24 [3]. To support this, it is necessary to understand factors that influence the behaviour of staff responsible for implementation.

The present qualitative study therefore aims to identify factors which influenced healthcare professionals’ implementation behaviour within the CURE project pilot site. The identification of these factors will facilitate learning to support recommendations for nation-wide roll-out of NHS funded tobacco dependence treatment services at scale.

## Methods

### Study design

Ethical approval for this study was granted from Northumbria University Faculty of Health and Life Sciences (ref 21,358). This research was underpinned by a social constructionist/relativist, descriptive approach and used qualitative, semi-structured one-to-one telephone interviews to explore factors influencing CURE implementation behaviour within the pilot site. Existing theoretical frameworks were used to support identification of barriers and facilitators. The Capability, Opportunity and Motivation for Behaviour (COM-B) model [9, 10] suggests engagement in a behaviour is determined by physical and psychological capability (e.g. physical skills, knowledge), physical and social opportunity (e.g. environment, social norms) and automatic and reflective motivation (e.g. habits, beliefs). The COM-B model sits at the hub of the Behaviour Change Wheel (BCW) [10], a systematic approach for developing and evaluating complex interventions. Application of the COM-B model can therefore facilitate intervention development or refinement [11–13]. For a more granular identification of factors known to influence behaviour and behaviour change, COM-B domains also map to the Theoretical Domains Framework (TDF) [14–16]. The TDF summarises 14 broad domains which explain behaviour in relation to ‘knowledge’, ‘beliefs about consequences’, ‘beliefs about capabilities’, ‘skills’, ‘environmental context & resources’, ‘social influences’, ‘memory, attention & decision processes’, ‘behavioural regulation’, ‘emotion’, ‘social or professional role/identity’, ‘optimism’, ‘intentions’, ‘goals ‘and ‘reinforcement’ [14, 15]. Both the COM-B and TDF are frequently employed to facilitate identification of barriers and facilitators to implementation behaviour [17–23].

This study and related findings are reported in line with the Standards for Reporting Qualitative Research (SRQR) checklist from the EQUATOR Network website.

### Context

The CURE project was piloted within a major acute teaching hospital in Greater Manchester, with approximately 950 beds. As well as routine acute care trust service provision for the local population, the hospital specialises in cardiology, cardiothoracic surgery, heart and lung transplantation, respiratory conditions, burns and plastics, cancer, and breast care services. The CURE pilot phase launched on 1st October 2018, running until 31st March 2019. Following this, the model was fully implemented into service at the pilot site and is currently in various stages of roll out within a further six hospitals across the county.

### Participants

A key contact was identified through Public Health England (PHE), who provided a list of potential participants (i.e., decision makers, senior management and clinical staff working across primary care, secondary care and public health, who had been involved in the delivery and/or implementation of the CURE project pilot). Participants were purposively sampled to access a range of experience relating to the implementation of the project. Participants were approached individually via email and invited to take part in the study. They were also provided with a participant information sheet explaining the purpose of the study. Due to the remote nature of the interviews, consent forms were sent and returned via email to those who opted to participate, prior to an interview taking place. If this was not possible, audio recording of consent was obtained and recorded separately from the interview audio recording. Both forms of consent were approved by Northumbria University Faculty of Health and Life Sciences Ethics Committee.

In total, 49 stakeholders were approached by the research team and 10 agreed to participate in interviews. Respondents’ professional background spanned core CURE management and specialist nursing staff, pharmacy, primary care, and public health. Of those who did not participate, there were 35 non-respondents and one individual who declined to take part as they did not have relevant experience of implementing CURE. Three contacts expressed difficulty finding suitable time to participate given staff shortages and retraining related to the COVID-19 pandemic.

### Data collection

One-to-one, semi-structured telephone interviews were conducted by an experienced researcher (AW), between February and April 2020. Telephone interviews have previously been associated with increased privacy and flexibility, thus encouraging disclosure [24]. The researcher was not previously known to participants, but introductions and brief communication had taken place via email, prior to interviews taking place. Topic guides were informed by TDF domains and were iteratively developed in response to feedback from early participant interaction. Topics included barriers and facilitators to delivering and/or implementing CURE, staff training and engagement, attitudes and beliefs towards the CURE project and service impact. Interviews lasted an average of 48 min (range 35–68 min). Full copies of the interview guides are available in additional file 1.

### Data analysis

All interviews were audio-recorded and transcribed verbatim. Transcripts were coded and analysed using the Framework method [25] to develop a thematic framework of barriers and facilitators to delivery and implementation of the CURE model. Firstly, three members of the research team (AW, AR and AH) independently coded the first three participant transcripts line-by-line. These codes were then mapped to TDF domains through consensus discussion to form the initial coding framework. This coding framework was applied on a flexible and iterative basis to all remaining transcripts to form a final thematic framework of factors influencing implementation of CURE (AW). If codes were linked to more than one area, they were categorised under the most relevant domain via discussion throughout the analysis process (AW, AR, VM, AH). The final framework was discussed amongst the research team and again applied to the whole dataset to ensure themes were reflective of participant responses. Identified TDF domains were then mapped to the COM-B model to support future intervention development.

## Results

### Participants and descriptive data

The 10 individuals who participated represented both those working within management/leadership roles, of varying levels of seniority, involved in the CURE pilot project team (*n* = 7) and Specialist Smoking Cessation Nurses (*n* = 3). Participants’ involvement in CURE spanned from the initial planning stages to the present day. Table 1 provides an overview of participant characteristics. Further breakdown of demographics by participant is not supplied to maintain anonymity.

**Table 1:** Overview of participant characteristics

	Total
**Age**	
Mean (SD)	44.67 (11.20)
Min-Max	31–62
**Sex [n (%)]**	
Female	5 (50)
Male	5 (50)
**Ethnicity [n (%)]**	
British	10 (100)
**Role [n (%)]**	
Management/leadership^a^	7 (70)
Deliverer^b^	3 (30)

^a^Refers to 2 core CURE team members, 2 steering group members with primary care and public health experience, 1 Primary care, 1 Pharmacy and 1 Public Health Specialist

^b^Refers to CURE specialist smoking cessation nursing staff

### Main results

In order of frequency (number of transcripts in which a domain occurred) and elaboration (number of codes within a domain), TDF (and COM-B) domains influencing the implementation of CURE were, ‘environmental context and resources’ (physical opportunity), ‘social influence’ (social opportunity), ‘goals’, ‘professional role and identity’ and ‘beliefs about consequences’ (reflective motivation),‘reinforcement’ (automatic motivation), ‘skills’ and ‘knowledge’ (psychological capability). A coding framework is available in additional file 2. Findings relating to each domain are summarised below. Some key illustrative quotes are provided within the text, with further examples provided in Table 2. Links between themes are cross-referenced in the text.

**Table 2:** Barrier and facilitators to implementation of the CURE model, with illustrative quotes

COM-B domain	TDF domain	Sub-themes	Barrier/ facilitator/mix	Illustrative quotes
**Opportunity**				
**Physical Opportunity**	**Environmental Context and Resources**	*Integration with the wider health care context*	B	*We kind of…mistakenly thought at the outset, was we get the CCG at the table then we’re kind of talking to everybody out in the community. But it’s really important to get out and see local GPs because they’re all little independent businesses. You know, they are joined by the CCG, but they’re independent practices. So it is, you can talk to a CCG and might have a view of the locality, but you do also have to get into individual GPs, and that’s quite hard to do.* -P1, management.
		*Staffing Resources*	M	*…we’re only just catching up on [follow-up calls] now and we’ve had help from an admin person who screens the calls first, see if patients want to be followed up. So that’s working quite well at the moment. So, she follows them up. Anybody that wants to be seen by a specialist nurse she refers them on to us. Well, it’s taken lots of pressure off really as well.* -P6, deliverer. *I think [the project manager] was the instrument behind all of that. She was just incredible. So, one incredible person kind of changed the whole thing around.* – P10, management.
		*Secondary-care context*	M	*We were in a crowded office with two or three other teams. We had two chairs between five of us. Two computers between five of us. And not a lot of space and you couldn’t make phone calls and we were disturbing them, they were disturbing us, and it was just terrible. So, we’ve got this nice big office now which has now become full*. - P6, deliverer. *There are situations where a patient might be in a busy bay of four patients, so it’s not always ideal in that sense, but you do the best that you can.* – P5, deliverer.
		*Availability of CURE related knowledge and training.*	M	*…in the background there’s been training for different professionals in the hospital so that it becomes everybody’s responsibility to see smoking cessation as part and parcel of everyday conversations, everyday delivery. And so that has gradually improved and improved. As a team people become more and more aware of us, so there’s things out there on the intranet every so often reinforcing what the CURE project is about. So, 8, 9 months ago I might have gone on a ward and someone said, when I said who I am, why I’m there, oh we’d not heard of the CURE project. You wouldn’t expect that to happen now.* – P5, deliverer.
		*CURE Branding*	F	*It starts at the basics, like a logo, and you start to realise the power just something of a simple logo. It started to build momentum behind it and started to get seen and started getting recognised. And so, what starts as something quite basic then really comes up and through that process becomes increasingly more complex and impactful* - P1, management.
		*Flexibility of service*	F	*Even though we have set clinic times, like we do morning clinics and afternoon clinics, if a patient can’t make those, I can say right [when] can you get to the hospital? They say well I can get there for ten. So quite often we’ll make an appointment to see them in a Costa coffee or there’s a Subway whatever it is* – P4, deliverer.
**Social Opportunity**	**Social Influences**	*Peer support*	F	*[The clinical and nurse leads] being a doctor and a nurse that worked in that hospital itself would go and present at every single training session that there was available. And obviously I would get [the clinical lead] to go to the doctor ones and I’d get [the nurse lead] to go the nurse ones, because I think having peer to peer explanation, I think, can be a lot stronger than sometimes*. – P7, management. *I introduce certain things myself…within the team, of things that I’ve done before. So, we do share knowledge as well.… [I send] information over to other colleagues, less experienced colleagues who then get regular updates on that*. – P5, deliverer.
		*Changing the culture of smoking cessation*	B	*One of the biggest roles [the clinical lead has] is then getting [CURE] out into all the places around the hospital, talking to as many clinicians as possible...whether it’s a junior doctor training session, whatever it is any session where we can discuss with clinicians about prescribing and changing how they…really changing their view of what they do with the smoker and moving away from the traditional view of smoking’s a lifestyle choice* – P1, management.
**Motivation**				
**Reflective Motivation**	**Goals**		M	*It’s quite hard to keep that level [of promotion] up and not let it dwindle, because in a years’ time you’re going to have a whole new set of junior doctors. And so, you need to do the same thing again. […]. But that is a challenge, keeping the level of enthusiasm and message up over time.* – P1, management. *Still [CURE has] never been modelled in a way to say that because a person’s stopped smoking and he hasn’t had any admissions, he hasn’t seen his GP or she hasn’t been having any long term problems. Does that make sense? So even though we know that they would stop, and just because they’ve stopped, well how can you say that it actually has an impact on our system? I think that was the hardest thing to sell.* -P10, management.
	**Professional role and identity**		M	*The culture is still a problem, so again, whose job it is. ‘This is not my job’. So, the problem was always tobacco addiction treatment is not my job. ‘The skill that I have is not really suited for this’*. - P10, management.
	**Beliefs about consequences**		F	*To me that is the most valuable thing, that you’re improving patients’ lives.* - P2, management.
**Automatic Motivation**	**Reinforcement**		M	*I think the vast majority of people you speak to it is like ‘oh this is brilliant, my practice has changed just from a 30 min talk’, which makes it a really rewarding thing to do in the future.* – P1, management. *Most [patients] do want to quit. You want to see the benefits of that and yeah, that keeps you going really. And also, when they do manage to quit, we become so pleased. I’ve had patients that say even whatever they spend buying cigarettes, tobacco, each week they put money in the jar and it’s that financial benefits as well. But I think it’s the main that their long-term health benefits’.* – P4, deliverer.
**Capability**				
**Psychological Capability**	**Skills**		M	*I suppose through my background and experience I have a way of working with people that’s worked for a long time* – P5, deliverer.
	**Knowledge**		F	*I think the proof of concept was the main argument, I think. And because within that was a financial argument. About reducing readmission rates and things and that’s music to the ears of commissioners because most of…roughly two thirds of the CCG’s money disappears into secondary care. So, anything that improves patient lives but also reduces admissions, the readmission rate and things.* – P2, management.

### Environmental context and resources (physical opportunity; mixed barrier/facilitator)

All participants discussed the contextual environment in which CURE existed in-depth, suggesting this domain had influence in both helping and hindering successful implementation. Sub-themes are presented below to detail the breadth of this domain.

#### Integration with the wider healthcare context (barrier)

Considering the post-discharge support offered to patients, participants felt there was a need for CURE to be integrated with primary care, community stop smoking services and pharmacies. However, across all interviews, it was suggested that integration with the wider healthcare context was the most prominent ongoing barrier to CURE implementation and delivery. Participants suggested a lack of funding for community support and a delay in involving independent GP practices during the planning stages exacerbated these challenges and contributed to difficulties working cohesively across contexts. Most commonly, participants highlighted the variable levels of smoking cessation support across local areas, which presented distinct challenges to ensuring consistent post-discharge care for patients. Within the pilot, this was resolved by CURE nurses taking on responsibility for providing follow-on support, but this increased time pressures for the core team and required additional administrative support (See also *1.2 Staffing resources, 1.3. Secondary-care context).*

*‘…the variation in support after discharge across Greater Manchester is huge. So, we had to deal with that and that is probably the biggest ongoing challenge that there is’. –* P7, management.

#### Staffing resources (mixed barrier/facilitator)

Nine participants highlighted the need to have adequate additional staff available to deal with the complexities of implementation and delivery. This included a project manager (highlighted as a critical facilitator of implementation), a team of CURE specialist smoking cessation nurses and administrative staff to support day-to-day delivery. In the early stages of the pilot, it was suggested limited numbers of CURE nurses and administrative staff were a barrier to CURE delivery. Participants described how the addition of more staff, particularly administrative support, later in the pilot phase facilitated the day-to-day smooth running of the project and allowed nursing staff to focus on supporting patients. Whilst staffing levels became less of a problem in the pilot site over time, a participant working within primary care suggested that the need for additional staffing within the community had been overlooked and was a contributor to the variability in post-discharge support.

*‘CURE obviously recruited nursing people so they had their built team around that, I think, which is essential because it brought bodies in to do something and they would do it and it would work. And that is always then challenge that was given to us, that you guys have got a team to do this, and if you send [patients] home [primary care] don’t have any teams. You’re relying on the existing teams to do it. So just offering money won’t help. We need bodies and we need people and we need the funding to do it.’* – P10, management.

#### Secondary-care context (mixed barrier/facilitator)

Eight participants highlighted the influence of the secondary-care context in which CURE is delivered. One participant identified a positive aspect of working within the existing hospital environment; the proximity of clinicians and pharmacy staff facilitated communication between groups and resulted in more efficient problem-solving. Existing data systems within the hospital (e.g., the Electronic Patient Record system) had been modified to document, review and monitor CURE-related patient and performance data, which also facilitated day-to-day delivery. However, participants also emphasised barriers to delivering CURE within a busy hospital environment. A lack of private office space and available computers, as well as limited availability of NRT on wards, had posed initial challenges to delivery in the early stages of the pilot. Whilst these barriers had been addressed over time, delivery staff also highlighted ongoing challenges delivering smoking cessation advice and support within the context of other urgent medical issues and a general lack of privacy on typically busy and open wards.

#### Availability of CURE related knowledge and training (mixed barrier/facilitator)

Eight participants referred to the availability of CURE-related knowledge and training in influencing implementation of the project. CURE nurses described a comprehensive training package for the core team, including mandatory online training modules and shadowing of more experienced staff. These online training modules were also made freely available via the CURE website, and uploaded to the Trust’s online training hub, for any health professional to access. In addition, CURE nursing and clinical leads ran information sessions at lunchtimes to engage wider hospital staff. Participants emphasised that the broad availability of CURE information and training aimed to encourage culture change around smoking cessation (See also *2.2 Changing the culture of smoking cessation*) and was successful in increasing understanding and awareness of the project. However, three participants suggested that training for deliverers and the wider team of individuals involved in CURE could have been made available earlier, to engage staff and facilitate implementation from project launch.

#### CURE branding (facilitator)

Five participants strongly emphasised the design and packaging, or branding, of CURE as a facilitator of implementation and delivery. Including a graphic designer from the early planning stages had ensured strong, professional, and eye-catching marketing materials which were used as tools to increase interest and awareness across all levels of hospital staff.

*‘I think the branding of CURE has been so pinnacle in getting people to recognise it and then buy-in*.’ – P7, management.

#### Flexibility of service (facilitator)

Six participants referred to the flexibility of the intervention as a strength of the project. Although working to a core service specification, there was an ability to amend CURE in line with patient need and available resources. In practice, this flexibility allowed deliverers to arrange extra appointments if participants requested additional support, as well as the ability to arrange outpatient appointments outside of set clinic times, and in more informal locations around the hospital in line with patient preference. This flexibility was in line with the CURE team’s underpinning commitment to offering patients personalised CURE support (See also 4*.0 Professional role and identity*).

### Social influences (social opportunity; mixed barrier/facilitator)

#### Peer support (facilitator)

Nine participants discussed how peer support enabled CURE implementation and delivery within the hospital environment. CURE nursing staff highlighted teamwork amongst peers through sharing of information and social support, which helped to overcome general challenges during day-to-day delivery of the project. Most commonly however, CURE champions, or peer leaders, were emphasised amongst both delivery and managerial staff as a critical facilitator of implementation, which encouraged culture change and staff engagement (see also *2.2 Changing the culture of smoking cessation*). It was suggested that observing familiar clinical and nurse leads, presenting and promoting CURE, motivated buy-in at both management and delivery staff level.

*‘[The clinical lead] was an incredibly persuasive individual, and he, for me, not only when he was selling it within the hospital, and certainly within this group, his leadership was incredible.’* – P2, management.

#### Changing the culture of smoking cessation (barrier)

Five participants suggested there was a need for culture change when implementing CURE. Participants perceived an underlying culture, within the wider healthcare context, of smoking cessation being viewed as a lifestyle choice (i.e., rather than tobacco dependence), which in turn could potentially cause problems in accessing funding and resources in the earlier stages of implementation, as well as difficulties with the prioritisation of smoking cessation amongst clinicians. Problematic cultural beliefs around smoking cessation were also highlighted as an ongoing barrier to integrating with primary care, where treatment and support were traditionally expected to be provided via specialised community support, rather than general practitioners (See also *1.1 Integration with the wider healthcare context, 4.0 Professional role and identity).*

*‘…if the person who’s making the decision still sees smoking as a lifestyle choice, they won’t stump up funding to treat it. And I know that’s a really hard thing to say, and I’m not saying it happens anywhere in particular, but as in we do have those challenges as well as personal opinion of people as to whether it’s important or not can create challenges*.’ – P7, management.

### Goals (reflective motivation; mixed barrier/facilitator)

Seven participants discussed the importance of setting, and working towards, shared goals as a facilitator of CURE implementation. Overall, there was an overarching aim across core CURE management and nursing staff to promote the benefits of the CURE project and encourage ‘buy-in’ from senior decision makers and healthcare professionals. At management level, this involved collaborative discussions with decision makers to highlight how CURE (and prioritising tobacco dependence treatment) aligned with existing hospital goals and priorities. CURE leads and specialist nursing staff also promoted the protocols at the heart of CURE (i.e., offering treatment and support to all smokers in secondary care) amongst the wider team of hospital staff. However, there were concerns over the ability to sustain the required level of enthusiasm and promotion of CURE long term, particularly due to constant rotation of junior staff. To extend ‘buy-in’ across the wider healthcare context (see also *1.1 Integration with the wider healthcare context*) it was also suggested that there was an ongoing need to identify clear pathways to evaluation, that evidence the success of the project, from the outset. However, this required specific expertise in evaluation methods which was not immediately available during the planning stages of the pilot.

### Professional role and identity (reflective motivation; mixed barrier/facilitator)

Nine participants suggested perception of one’s professional role/identity could facilitate, or conversely hinder, implementation of the CURE project. Participants discussed an underlying conflict over who was responsible for smoking cessation related treatment and support, as it was not traditionally part of medical staff’s role or professional identity to provide this. Whilst CURE champions/peer leaders were believed to be effective in changing views towards medical staff’s role, it was suggested that this was an ongoing barrier to engaging individuals within the wider healthcare context.

*‘For a long time, it’s been, well, this is someone else’s [role], we’ve never seen it as doctors or prescribing nurses. We’ve not seen it as our role to be really proactive in smoking.’ -* P1, management.

However, interviewees expressed a commitment to offering and encouraging patient choice as an integral aspect of the project, which aligned with CURE principles and thus facilitated staff engagement and delivery. Whilst having to consider available funding to support drug treatments, managerial staff suggested an important part of their CURE role was to offer options to the patient, particularly regarding NRT and follow-up care. Deliverers also emphasised that CURE was a patient-led project, whereby staff supported individuals to come to a shared decision on whether they were ready to stop smoking (or not).

### Beliefs about consequences (reflective motivation; facilitator)

Seven participants expressed positive beliefs surrounding the CURE project. Participants believed that the inclusion of behavioural and psychological support into the model facilitated rapport building with patients, which in turn increased patient engagement in the project. This belief motivated staff to implement CURE as they felt there was a real opportunity for the project to offer targeted and tailored support. In turn, participants expressed a strong belief that CURE was therefore an effective solution in treating tobacco dependence and smoking related ill-health, thus improving patients’ lives.

*‘I do believe in what I’m doing. I mean I had a lot of positive experiences in the past with people changing their life around, so difficult not to believe in it and be enthusiastic about it, you know’* – P5, deliverer.

### Reinforcement (automatic motivation; mixed barrier/facilitator)

Eight participants referred to the influence of rewards and incentives to deliver CURE. Following the difficulties integrating the service into primary care, three participants mentioned that financial incentives were offered to GPs, to encourage NRT prescribing. However, upon piloting this approach, many GPs did not complete reimbursement documents. The reasons for this remain unclear, though it was suggested that the financial incentive became of less importance once individual practitioners had an awareness and understanding of the service. However, participants across management and delivery roles suggested reflection on intrinsic rewards motivated individuals to implement CURE. These rewards ranged from changing other’s practice, to observing health and/or financial benefits of smoking cessation for patients.

### Skills (psychological capability; mixed barrier/facilitator)

Seven staff referred to the influence of their own and/or others existing skills and experience on implementation. Deliverers suggested previous experience and skills supporting smoking cessation, and using existing data systems, were beneficial. Conversely a lack of experience in these areas, particularly in relation to the use of IT systems, may hinder day-to-day delivery. For managerial staff, previous experience of asset-mapping, commissioning services and working within the locality was valuable and facilitated identification of available resources during the planning stages.

### Knowledge (psychological capability; facilitator)

Three participants suggested that acquiring knowledge of supporting evidence (initially based around the effectiveness of NRT and the Ottawa model) was particularly important in encouraging buy-in from senior management and decision makers (see also *3.0 Goals*).

*‘So, reading all the papers on the effectiveness of the drugs that are given for tobacco addiction [was important]. So, all that needed to be done so that we are a voice which is not just passionate but is well educated and informed’.* – P10, management.

## Discussion

### Principal findings

This qualitative study aimed to identify barriers and facilitators to implementation of the CURE project in the pilot site. Findings were presented in the context of the TDF [15, 26], and further mapped to the COM-B model [9, 10], with eight TDF domains identified as influential to project implementation: ‘environmental context and resources’ (physical opportunity), ‘social influence’ (social opportunity), ‘goals’, ‘professional role and identity’ and ‘beliefs about consequences’ (reflective motivation), ‘reinforcement’ (automatic motivation), ‘skills’ and ‘knowledge’ (psychological capability). These findings demonstrate the complex combination of influences on healthcare professionals’ behaviour [16]. Findings were informed by a variety of stakeholder experiences (as participants worked across secondary care, pharmacy, primary care and public health). These experiences related to all phases of implementation from the initial planning stages to full implementation.

Despite the wide range of experience, the most commonly identified barriers were consistent across participants. These referred to challenges integrating CURE within the existing healthcare environment. In addition to this, CURE nursing staff commonly highlighted time pressures, resource availability and the secondary-care environment as barriers to delivery. This is consistent with previous research which also demonstrates the potential challenges presented by health professional’s workplace environments and available resources during intervention implementation [17, 27, 28]. In the present study, early engagement with stakeholders, ensuring adequate staffing resources and wide availability of CURE related knowledge and training had the potential to reduce some of these barriers.

Several facilitators were also identified throughout the interviews. Across participants, the most commonly emphasised facilitator referred to the importance of peer support and leadership i.e., through CURE champions. Past evidence suggests clinical champions can help prioritise tobacco dependence treatment within the hospital environment and encourage staff engagement [29]. Observing clinical champions support an intervention can therefore elicit behaviour change at the individual and ultimately organisational level [30–32]. The present findings give insight into how this process occurs, suggesting champions facilitate knowledge exchange and influence changes to professional identity that are needed for the system-wide cultural shift CURE aims to achieve.

Some of the reported factors within the present study, such as competing priorities, resource availability and training opportunities, are commonly identified as challenges to intervention implementation within secondary care settings [33, 34]. However, this study also highlights the importance of less commonly discussed factors on health professional behaviour. For example, flexibility within the service specification was highlighted as a facilitator of implementation which allowed CURE to be responsive and adaptable to both patient and hospital needs. Flexibility within a service specification has recently been identified as an important component of implementation across healthcare settings [35] and may increase adherence, acceptability and sustainability of hospital-based interventions [33]. Similarly, the inclusion of patient choice within CURE (e.g., related to NRT and follow-up support options) was also discussed as facilitator of implementation behaviour, as this aligned with participant’s professional identity. Evidence suggests that motivation to adopt and implement interventions is influenced by the degree to which an intervention aligns with healthcare professional’s identity [17, 18, 20, 21]. It would therefore be beneficial to explore how to integrate and emphasise these flexible and patient-choice focused aspects of the CURE project into wider roll-out of secondary care based smoking cessation initiatives, also considering how these aspects may need to be balanced with intervention fidelity [36].

As detailed above, our approach identified COM-B components relevant to implementation of the CURE project; when developing strategies for implementation, healthcare professionals’ psychological capability, physical opportunity, social opportunity, reflective motivation and automatic motivation are important to target. CURE’s existing implementation strategy content and potential mechanisms of action have been defined and described elsewhere [37] using the BCW [10]. Further application of the BCW [10] within this context links the COM-B components identified here to nine specific intervention functions (IF’s; i.e., ways in which the identified barriers may be addressed); Education, Persuasion, Incentivisation, Coercion, Training, Environmental Restructuring, Modelling and Enablement. As healthcare professionals’ capability, opportunity and motivation all had the potential to act as both barriers and facilitators to change, all nine IF’s may be considered to strengthen CURE’s existing implementation strategy. However, the suitability of these IF’s are dependent on the context of each specific barrier, the intervention content itself and intervention setting (i.e., secondary care) [10], thus selection of IF’s should take place in conjunction with stakeholders and/or those who have in-depth knowledge of the secondary care setting. This approach would also be valuable in identifying feasible policy categories, associated with these IF’s (Guidelines, Environmental/Social planning, Communication/Marketing, Legislation, Service Provision, Regulation, and Fiscal measures) [10] to further develop and refine suitable and feasible implementation strategies on a wider scale.

### Implications for practitioners and policymakers

As indicated above, the CURE project aims to medicalise tobacco dependence and move beyond the view of smoking behaviour as a lifestyle choice [7, 8]. To achieve this aim, a national model should include strategies to encourage staff engagement and culture change. Throughout the interviews with stakeholders, it was suggested that clinical champions were particularly effective in engaging and motivating staff members across all levels of seniority and could be influential in changing unhelpful cultural beliefs around smoking cessation. As such, clinical champions should be identified for each site as early as possible. Champions have also been shown to enhance long-term sustainability of the OMSC, suggesting benefits of this approach may extend beyond implementation [29]. As discussed above, the focus on patient choice and shared decision making within CURE should also be emphasised when aiming to engage stakeholders, as participants indicated this approach was in line with their professional identity and also aligns with current NHS guidance [3, 38, 39]. Marketing materials should also be considered a priority in engaging healthcare staff when implementing a CURE style model in new sites. Participants indicated that both the design and wide presence of CURE posters, screensaver, cards and pens had been vital in increasing interest and familiarity surrounding CURE. Signposting to freely available and easily accessible training materials, from the earliest possible stages, also ensures the wider hospital team feel involved and informed in implementing CURE within their own workplace.

Although CURE is secondary care-led, increased integration within the wider healthcare environment has the potential to maximise impact. Effective patient discharge pathways rely on collaboration with general practitioners, community pharmacists and stop smoking services. It is therefore integral to ensure efforts to engage healthcare professionals and decision-makers go beyond hospital-based teams. Managerial staff highlighted this as one of the most challenging aspects of implementation and suggested early engagement and collaborative discussion were the most effective ways of engaging relevant groups. Engagement and discussion with Local Medical Committees and Medicine Optimisation Services should therefore be arranged from the very initial stages of planning, to encourage collaborative working and implementation of CURE across settings.

### Strengths and limitations

This study is the first to qualitatively explore barriers and facilitators to the implementation of the CURE project. Considering factors through the lens of the TDF and COM-B allows for the identification of both internal and external factors which are known to influence behaviour change, and thus allows for targeted intervention development to support future implementation [10, 16]. Beyond the CURE project, these findings also contribute important insight into factors which influence behaviour change in healthcare professionals and therefore may have value across a variety of related contexts. The use of established theoretical frameworks also facilitates efficient translation to policy and practice [10].

Despite its strengths, there are two main limitations of the findings reported here. Firstly, the present research was undertaken between February and April 2020, during the increasing prevalence of COVID-19 cases within the UK. From March 2020 onwards, COVID-19 was declared a pandemic [40] and frontline care was prioritised [41]. As such, there were significant recruitment challenges which resulted in a smaller than anticipated sample and under-representation of both specialist and non-specialist delivery staff. The lack of delivery staff within the sample may therefore have resulted in findings that are predominantly focused on barriers and facilitators to implementation at an administrative, rather than delivery, level. Given the low uptake of invitations to participate, it is also possible that views may be biased towards those that held favourable opinions of CURE. As such, the results reported here may not be fully representative of the wide variety of stakeholders involved in delivering CURE. For example, the confidence of healthcare professionals is likely to influence delivery of an intervention [27, 42] yet this was not identified as a relevant domain within the current study. Findings should therefore be viewed with these limitations in mind.

### Future research

This research presents several avenues for further research. As mentioned above, the present study was conducted during the COVID-19 pandemic, which had an impact on the availability of healthcare professionals to participate. Future related work should prioritise the collection of views and experiences from stakeholders involved in the delivery of CURE e.g. specialist CURE nurses, as well as junior doctors, nursing staff and general practitioners who either refer to the core CURE service and/or are able to offer community-based support upon discharge. The inclusion of these stakeholder groups would allow for additional insight surrounding key barriers related to delivery of the intervention, such as time pressures, access to pharmaceuticals and variability in post-discharge support.

The factors identified within this research should be used to inform healthcare professional behaviour change strategies to support future implementation of secondary care-based tobacco dependence treatment and support. As indicated above, further application of the BCW [10] identifies nine specific IF’s and seven policy categories that have the potential to address barriers identified through this research and can therefore strengthen CURE’s existing implementation strategy. To refine selection of suitable IF’s and policy categories (i.e., to the context in which they will be implemented) stakeholder workshops should be conducted involving health care professionals, NHS England Improvement, PHE Behavioural Insights, PHE Tobacco team, leading academics in the field and other stakeholders [10]. This approach would allow for the co-development of acceptable, practical and feasible evidence-based recommendations to support wider rollout of secondary-care based tobacco dependence treatment and address the barriers highlighted within this research.

There is also an opportunity to investigate the perspectives of healthcare professionals in different settings and other roll-out sites, particularly as healthcare delivery changes and adapts in light of the COVID-19 pandemic.

## Conclusion

In line with the NHS Long Term Plan [3], development of a service specification for a national stop smoking model based on CURE is due to begin in Autumn 2020. This study highlights barriers and facilitators to implementing the CURE project pilot and provides evidence which can inform the development of theoretically based implementation strategies to support secondary care-initiated tobacco dependence treatment. The application of the TDF and COM-B model within this context demonstrates the broad range of multi-level influences on healthcare professionals’ implementation behaviour. As such, taking a whole-systems approach and working collaboratively with healthcare professionals and decision-makers across settings and services could maximise the impact of the intervention in encouraging a widespread culture shift in smoking cessation treatment and support.

## Supplementary Information

**Additional file 1.** Topic Guides.

**Additional file 2.** Coding Framework.

## Data Availability

Due to ethical concerns and the type of consent provided from participants, the qualitative transcripts underlying this research cannot be made publicly available. Sharing this data publicly may compromise anonymity of participants due to the experiences that were shared throughout interviews. Qualified researchers may contact the authors of this study (angela.rodrigues@northumbria.ac.uk) for inquiries regarding data access.

## Abbreviations

BCW: Behaviour change wheel
COM-B: Capability, opportunity, motivation for behaviour
CURE: Conversation, understand, replace, expert and evidence based treatments
IF’s: Intervention functions
NHS: National Health Service
NRT: Nicotine replacement therapy
OMSC: Ottawa Model for Smoking Cessation
PHE: Public Health England
TDF: Theoretical Domains Framework
