# Supplementary material for: Barriers and facilitators to implementing the CURE stop smoking project: a qualitative study
Source: BMC Health Serv Res. 2021 May 20;21:481. doi: 10.1186/s12913-021-06504-2 (PMC8136754; doi:10.1186/s12913-021-06504-2)
Supplement: Supplementary file 1 — Additional file 1. Topic Guides. [file 12913_2021_6504_MOESM1_ESM.docx]

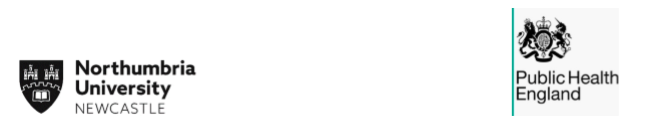


**CURE Model INTERVIEW TOPIC GUIDE (Management/Leadership)**

Thank you for agreeing to take part in this interview. We are inviting a number of health care professionals involved in implementing the CURE Model in Greater Manchester so I am pleased you are able to speak to us today. We are interested in your behaviour (in implementing the CURE Model), your perception of the impact on patients and ways to help more people in the future.

**A. YOUR ROLE/RESPONSIBILTIES**

- **Could you give me an overview of your current occupation and responsibilities?**
- **How are you involved in implementing the CURE programme?**
  - How long have you been involved in overseeing the implementation of CURE?
  - Has your involvement in the programme changed over time? If so how/why?

**B. IMPLEMENTING CURE – PRE-LAUNCH:**

- **What did you need to do prior to the launch of the CURE programme?**
  - What did you need to know to be able to do this?
  - Who else was involved in the planning stages of CURE implementation, to your knowledge?
  - Have you had any previous experiences of implementing or overseeing interventions or support around quitting smoking?
  - If so, what are the differences between your previous experience and implementing the CURE programme?
- **What resources did you need to facilitate implementation in the earlier stages? How did you access these resources? (e.g. I.T, staffing, finance)**
- **Have you felt the need to change/modify the CURE programme in any way at a? If so, can you tell me more about how you did this and why?**
- **What challenges did you encounter during the planning process? At what stage did these occur?**
  - How did you manage these challenges? What helped to overcome them?
  - What social and cultural factors are there to consider when planning this programme? (workplace culture, language barriers, etc.)
  - What challenges arose with finding suitable venues for clinics? (if applicable)
- **How did you incorporate the planning of the CURE programme into your everyday practice? How did this fit with your other responsibilities?**
  - How did you manage the different tasks associated with the planning of CURE?

**C: IMPLEMENTING CURE - POST LAUNCH:**

- **Did the initial launch go as planned? What do you feel this was this due to?**
  - What did you hope to achieve when launching the programme?
- **What resources are necessary to implement the programme in practice? How do you ensure those resources are available?**
- **Were there any challenges of implementing this model into practice? When did these challenges occur? (i.e. the initial phase, or during/throughout pilot of the programme or following implementation?)**
  - What helped to manage these challenges?
  - How did these challenges change over time? (i.e. throughout pilot phase; refer to previous two questions if appropriate).
  - How well does CURE integrate with existing service? How easy/difficult has it been and why?
- **How do you manage to incorporate the oversight of CURE into your everyday practice (i.e. make it a habit)?**
  - Were there any challenges to fitting this in alongside your other responsibilities?
  - Are there procedures or ways of working that encourage successful implementation of the programme? If so, can you tell me more about these?
  - **Are you supported to oversee the CURE programme**? Does this allow you to do so as intended?
  - Are there competing tasks and time constraints when overseeing the programme? If so, what are these?
  - How could your role in overseeing the programme be better facilitated?
  - Did you need to decrease/increase involvement in response to specific timepoints/milestones/events?
- **Have any adaptations been made to the CURE programme, following initial planning or implementation? If so, how/why?**
  - How does the setup of your workplace/organisation influence implementation of the programme? (i.e. does it help/hinder or result in the need for adaptations).
  - How could the overall implementation of the programme be improved?
- **Are there any issues/barriers to the effective implementation of the CURE programme?**
  - How confident are you that the problems of implementing the CURE programme will be solved in the future?

**D: STAFF TRAINING/ENGAGEMENT**

- **How do you ensure staff are motived to implement/deliver CURE as intended?**
  - **How do you encourage involvement or support from senior management?**
- **Have you been involved in organising staff training? If so, can you tell me how you did this?**
  - Are you aware of any challenges when organising staff training? Has this changed over time?
  - How have staff engaged with training
  - **How could the training be improved?**
  - Are there any plans for refresher training? If so, how will this be delivered?
- **Do you receive any feedback from staff who deliver the CURE programme? If so what format is this in? What are their general thoughts?**
  - How confident are you in the skills and capabilities of those who deliver this programme?
  - Do you feel some professionals may be better suited to delivering CURE than others? If so, why? (E.g. required beliefs/attitudes/behaviours).

**E. ATTITUDES/BELIEFS TOWARDS CURE:**

- **Did you initially support the CURE programme? Why/ why not? (Personal interest/meeting NHS targets/priorities)**
  - Have your views on CURE changed since first becoming involved with the programme? If so how/why?
  - What, in your opinion, are the benefits of the CURE programme?
  - What are the disadvantages (if any) of the CURE programme?
- **Do you see the oversight of the CURE programme as a core part of your role?**
  - Is the implementation of the CURE programme compatible or in conflict with your professional standards/identity?
- **Do you discuss CURE with colleagues? How do colleagues view the programme?**
- **What motivates you to implement CURE? (e.g. targets, health benefits, making a difference)**
  - What do you hope the programme will achieve?
  - How confident are you in your own skill and capability to oversee this programme?
  - Are you more confident in some areas (activities) than others?
  - How rewarding is your involvement with the programme?

**F. SERVICE IMPACT**

- **From your perspective, how well do patients engage with the programme?**
  - What do you think influences patients to engage with the CURE programme?
- **What do you think are the important barriers and facilitators for patients engaging with the CURE programme?**
  - How has your planning/oversight of the programme been affected by social and cultural factors of patients you wish to engage in the CURE programme?
  - What do you think helps patients to stay motivated?
  - Do you feel the programme works better for some people rather than others? If so, why?
  - **How could the programme be improved for patients?**
  - Do you think the information provided (to patients) in the CURE service is adequate?
  - Do you believe there are aspects of the CURE programme which are more valuable than others?
- **How do you ensure the programme is delivered as intended?**
- **Can you describe any unintended or unexpected consequences of the CURE programme? These can be positive or negative.**
- **How confident are you that the problems of implementing the CURE programme will be solved in the future?**

**G. SUMMARY**

**Are there any other factors influencing the implementation of CURE that we have not already covered?**


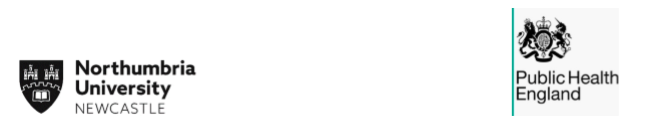


**CURE Model INTERVIEW TOPIC GUIDE (Delivery Staff)**

Thank you for agreeing to take part in this interview. We are inviting a number of health care professionals involved in implementing the CURE Model in Greater Manchester so I am pleased you are able to speak to us today. We are interested in your behaviour (in implementing the CURE Model), your perception of the impact on patients and ways to help more people in the future.

**A. YOUR ROLE/RESPONSIBILTIES**

- **Could you tell me a little about your current occupation and responsibilities?**
- **How are you involved in delivering/implementing the CURE programme? (e.g. what stage in patient pathway)**
  - How long have you been involved in the delivery of the CURE programme?
  - Has your involvement in the programme changed over time? If so how/why?

**B. DELIVERING THE PROGRAMME:**

- **What do you do as a deliverer of the CURE programme? (i.e. What behaviours? When? Using what systems? With who? How often?)**
  - What is your aim when delivering the programme?
  - What do you need **to know** to deliver this programme?
  - Is this a new behaviour? Have you had any previous experience of delivering interventions or support around quitting smoking?
  - If so, what are the differences between your previous experience and delivering the CURE programme?
- **Are there any specific resources you need to deliver your part of the programme? How do you access these resources on an ongoing basis?**
  - Who is involved in the delivery of the programme, to your knowledge?
  - In what setting(s) do you deliver the programme?
  - How suitable the current setting to deliver CURE?
- **How did you manage to incorporate the delivery of CURE into your everyday practice (i.e. make it a habit)?**
  - How do you decide what to do in each session? Do you remember everything you should / could show people?
  - Do you have to do any preparation before each session? If so, how do you prepare?
- **Have you made any adaptations or changes to how you deliver CURE since you first became involved? If so, how/why?**
  - Is the successful delivery of your part of the CURE programme dependent on other parts of the programme working well? If so, does this pathway work well?
  - Are you supported to implement the CURE programme? Does this allow you to do so as intended?
  - How does the setup of your workplace/organisation influence delivery of the programme? (i.e. does it help/hinder delivery or result in the need for adaptations).
- **What makes it difficult for you to deliver CURE?**
- **Are there any challenges/barriers to your effective delivery of the CURE programme?**
  - If so, what were these barriers and at what stages did they occur? (i.e. the initial phase, or during/throughout pilot of the programme)
  - What helped to manage challenges?
  - How did these challenges change over time? (e.g. throughout pilot phase)
  - Are there competing tasks and time constraints? If so, what are these?
  - How do you believe delivery of the programme could be improved?
  - How could the overall implementation of the programme be improved?
- **Are there procedures or ways of working that encourage the delivery of the programme?**
  - What factors make it easier for you to deliver CURE?

**C. SUPPORT IN DELIVERING CURE:**

- **Are the necessary resources available to those expected to deliver the CURE programme? What are the necessary resources?**
- **What training did you receive? (e.g. how much, in what format etc.)**
  - Did the training include watching other providers? If so, what did you learn?
  - Was this training enough?
  - Have you been involved in training/encouraging others to deliver the CURE model? If so, can you tell me a little more about how you have done this/your experience of this?
- **How could the training be improved?**
  - **What were the most challenging parts of the training (if any)?**
  - Do you need refresher training? Are you aware of plans for any further training, and how to access it?
- **Are you supported to deliver CURE? (e.g. leadership/management, colleagues, organisational culture). What has helped with this?**

**D. ATTITUDES/BELIEFS TOWARDS DELIVERY:**

- **What is your overall opinion of CURE? (i.e. in terms of benefits/disadvantages of the programme).**
- **How does delivery of CURE fit your other roles and responsibilities?**
  - Is the CURE programme in line with your professional identity?
- **How do your colleagues view the programme?**
  - Do you feel some professionals may be better suited to delivering CURE than others? If so, why? (E.g. required beliefs/attitudes/behaviours).
  - Do you discuss CURE with your colleagues?
- **What motivates you to deliver the programme (colleagues, patients, relatives, and friends, community, making a difference)?**
  - What do you hope to achieve by delivering the programme?
- **How confident are you in your skill and capability to deliver this programme?**
  - Are you more confident in some areas (activities) than others?
  - How well do you feel you deliver the programme as intended?

**E. SERVICE IMPACT**

- **In your experience, how well do patients engage with the programme?**
- **What do you think influences patients to engage with the CURE programme? /What do you think are the important barriers and facilitators for patients engaging with the CURE programme?**
  - Do you feel the programme works better for some people rather than others? If so, why?
  - Do you have any suggestions for how to change CURE so that more patients engage?
  - How is your delivery affected by the social or cultural background of patients you wish to engage with the CURE programme?
  - What do you think helps patients to stay motivated?
  - To what extent do emotional factors facilitate or hinder the delivery of the CURE programme?
- **Have you felt the need to change/modify the CURE programme dependent on the patients you are working with? If so, can you tell me more about how you did this and why?**
  - Do you believe there are aspects of the CURE programme which are more valuable than others?
- **Can you describe any unintended or unexpected consequences of delivering the programme? These can be positive or negative.**
- **How could the programme be improved for patients?**
  - Do you receive any feedback from patients after delivering the CURE programme? If so, what format is this in? What are their general thoughts?
  - Do you think the information provided (to patients) in the CURE service is adequate?
  - How rewarding is delivering this programme?

**F. SUMMARY**

**Are there any other factors influencing the delivery/implementation of CURE that we have not already covered?**
