# Supplementary material for: Barriers and facilitators to implementing the CURE stop smoking project: a qualitative study
Source: BMC Health Serv Res. 2021 May 20;21:481. doi: 10.1186/s12913-021-06504-2 (PMC8136754; doi:10.1186/s12913-021-06504-2)
Supplement: Supplementary file 2 — Additional file 2. Coding Framework. [file 12913_2021_6504_MOESM2_ESM.docx]

**Table 1. Coding framework - barriers and facilitators to implementation of the CURE project pilot.**

| **COM-B Domain** | **TDF Domain** | **Sub-theme** | **Barrier** | **Facilitator** | **Mixed barrier/facilitator** |
| --- | --- | --- | --- | --- | --- |
| **Opportunity** |  |  |  |  |  |
| **Physical Opportunity** | **Environmental Context and Resources** | Integration with the wider healthcare context | - Variability in post-discharge support - Integration with Primary care and external organisations |  | - Funding for all stages of support |
|  |  | Staffing resources |  | - Leadership in planning and oversight | - Staffing Resources |
|  |  | Secondary care context | - Time pressures - Access to pharmaceuticals - Office resources for delivery staff | - Data storage systems | - Delivery environment |
|  |  | Availability of CURE related knowledge and training. |  |  | - Availability of CURE related knowledge and training. |
|  |  | CURE Branding |  | - Cure Branding |  |
|  |  | Flexibility of service specification |  | - Flexibility of service specification |  |
|  |  |  |  |  |  |
| **Social Opportunity** | **Social Influences** | Peer Support |  | - Peer leadership - Teamwork |  |
|  |  | Changing the culture around smoking cessation | - Changing the culture around smoking cessation |  |  |
| **Motivation** |  |  |  |  |  |
| **Reflective Motivation** | **Goals** |  | - Managing competing goals and priorities - Identifying and evaluating CURE outcomes | - Adhering to a CURE service specification | - Promoting CURE |
|  | **Professional role and identity** |  |  | - Commitment to encouraging patient choice | - Professional identity |
|  | **Beliefs about consequences** |  |  | - CURE improves outcomes. - Personalised support improves patient engagement |  |
| **Automatic Motivation** | **Reinforcement** |  |  | - Reflecting on rewards of CURE involvement | - GP Incentives to engage with CURE |
| **Capability** |  |  |  |  |  |
| **Psychological capability** | **Skills** |  |  |  | - Previous experience and skills. |
|  | **Knowledge** |  |  | - Knowledge of supporting evidence. |  |
